# Hypohidrotic ectodermal dysplasia and physical exercise

**DOI:** 10.1186/1746-160X-8-S1-I12

**Published:** 2012-05-25

**Authors:** Johanna E Hammersen

**Affiliations:** 1 Department of Pediatrics, University Hospital Erlangen, Germany

## 

Because of their lack of sweat glands, individuals with hypohidrotic ectodermal dysplasia (HED) are assumed to be at risk of severe hyperthermia during exercise in a warm environment. If pediatric HED patients ask whether they may practice competitive sports, most physicians are hesitant to give recommendations other than swimming, which is unlikely to lead to life-threatening exertional overheating. To study the effects of physical exercise on HED patients more systematically and to determine levels of activity they tolerate and may engage in without health hazards, 13 boys and male adolescents with X-linked HED as well as age-matched healthy male controls were investigated during standardized exercise on a bicycle ergometer at ambient temperatures of 25°C and 30°C. Protective effects of evaporative skin cooling devices were evaluated at 30°C. Body core temperature during and after exercise, heart rate, performance, endurance, and serum lactate were analyzed. HED subjects experienced a significantly greater rise in body temperature after cycling than healthy controls, and their body temperature remained elevated longer. Maximum heart rates and lactate values did not differ significantly between HED and control groups. Application of skin cooling devices led to a clinically relevant attenuation of exertional hyperthermia in HED patients, and a previous tendency towards lower performance disappeared. This first systematic study of the effects of physical exercise on HED patients demonstrated a rapid and lasting body temperature increase in HED subjects after cycling, posing them at risk of exercise-induced hyperthermia and heat-related illnesses. External evaporative skin cooling attenuates exertional overheating in HED patients and may facilitate their participation in athletic activities and professional life.

